# Analysis of Defined Daily Dose (DDD) and antibiotic problems in Intensive Care Unit (ICU) patients

**DOI:** 10.1016/j.rcsop.2025.100688

**Published:** 2025-12-07

**Authors:** Yulistiani Yulistiani, Hargus Haraudi Barkah, Febriansyah Nur Utomo, Lucky Andrianto, Alena Putri Jathi, Nurrizky Grahitaning Putra Rohmaana Yudistira

**Affiliations:** a Department of Pharmacy Practice, Faculty of Pharmacy, Universitas Airlangga, Surabaya, Indonesia; b Department of Pharmacy, Universitas Airlangga Hospital, Surabaya, Indonesia; c Department of Anesthesiology, Faculty of Medicine, Universitas Airlangga, Surabaya, Indonesia; d Faculty of Pharmacy, Universitas Airlangga, Surabaya, Indonesia

**Keywords:** Antibiotic, Defined daily dose, DRPs, Resistance, Intensive care unit

## Abstract

The increasing prevalence of antimicrobial resistance (AMR) in Intensive Care Unit (ICU) is posing a significant challenge to effective infection management. This shows the need for quantitative evaluation of antibiotic use through the Defined Daily Dose (DDD) method, combined with Drug Related Problems (DRPs) analysis to support Antimicrobial Stewardship (AMS) programs. Therefore, this study aimed to analyze the quantitative use of antibiotic using DDD method and identify DRP in ICU patients at Airlangga University Hospital. A retrospective cross-sectional study was conducted using medical records of ICU patients from July to December 2022. Antibiotic consumption was calculated in DDD per 100 patient-days following WHO standards, while DRPs were identified using the Cipolle and Pharmaceutical Care Network Europe (PCNE) v9.1 classifications. A total of 125 ICU patients met the inclusion criteria, with a predominance of pneumonia (40 %) and sepsis (25 %). The results showed that the total antibiotic consumption was 91.54 DDD/100 patient-days, with ceftriaxone having the highest (31.57 DDD/100 patient-days). The most frequent DRPs were unnecessary drug therapy (20 %) and suboptimal therapeutic effects (23 %), mainly due to inappropriate indications and deviations from treatment guidelines. Antibiotic use in ICU was dominated by ceftriaxone, and the high rate of DRPs showed the need for improved AMS. This study showed that strengthening interdisciplinary collaboration, regular audits, and adherence to clinical guidelines were essential steps to optimize antibiotic use and minimize the risk of resistance.

## 1 Introduction

Antibiotic resistance or Antimicrobial Resistance (AMR) is a global health threat with a continuous increase in both developed and developing countries, across hospital and community settings. Therefore, the World Health Organization (WHO) has listed AMR as one of the top 10 global public health threats facing humanity.1., 2., 3. AMR occurs when microorganisms, particularly bacteria, develop mechanisms to survive antibiotic exposure, causing ineffective therapy. An estimation has shown that by 2050, AMR would cause approximately 10 million deaths annually, compared to the current global burden of cancer.^2^,^4^

In Intensive Care Unit (ICU), problems of antibiotic resistance is particularly alarming. The use of antibiotic in ICU is reported to be approximately 10 times higher than in general wards, due to the complexity of cases, immune system disturbances, invasive procedures, and high risk of cross-infection.^5^,^6^ Previous studies have shown that the irrational use of antibiotic in ICU significantly contributes to the occurrence of multidrug-resistant organisms (MDROs), leading to higher morbidity, mortality, and healthcare costs.^7^,^8^

Due to the increasing rate of antibiotic resistance, Antimicrobial Stewardship (AMS) programs have been implemented as control strategies. These include antibiotic classification using the AWaRe (Access, Watch, Reserve) framework, and quantitative evaluations of antibiotic consumption through the Defined Daily Dose (DDD) method.9., 10., 11 Several studies across different countries have reported significant variations in antibiotic consumption patterns in ICU. For example, in European ICU, broad-spectrum antibiotic such as ceftriaxone, meropenem, and piperacillin-tazobactam are frequently prescribed,^12^ while in Asian ICU, ceftriaxone and levofloxacin remain the dominant option.^13^, ^14^ These international disparities show the need for local quantitative evaluations to identify specific antibiotic use trends and potential misuse.

Most existing studies focus primarily on measuring antibiotic consumption without assessing the appropriateness of therapy. However, the integration of DDD-based quantitative evaluation with Drug Related Problems (DRP) analysis, such as using the Cipolle or PCNE v9.1 frameworks, remains limited, particularly in Indonesian ICU settings. This creates an essential knowledge gap in understanding how much antibiotic is used and the appropriate prescription. Therefore, this study aims to evaluate the quantitative use of antibiotic and identify potential DRPs among ICU patients in Airlangga University Hospital. The results are expected to provide essential insights to optimize AMS and reduce resistance risks at the local and national levels.

## 2 Materials and methods

This study used a retrospective cross-sectional design, with medical record data from patients admitted to ICU of Airlangga University Hospital between July and December 2022. The retrospective method was selected to allow the evaluation of antibiotic use patterns and DRP based on existing patients data within the defined study period. A purposive consecutive sampling method was used, where all ICU patients who met the inclusion criteria during the study period were added until the required sample size was achieved. This method ensured that all eligible cases representing the study population were analyzed without randomization.

Data were collected retrospectively from electronic medical records, including patients demographics, clinical diagnoses, and details of antibiotic therapy. Quantitative antibiotic use was evaluated using DDD per 100 patient-days as recommended by the WHO (2003).^10^ Identification of DRPs was performed using the Cipolle classification and the Pharmaceutical Care Network Europe (PCNE) version 9.1 criteria.

The data obtained were tabulated and analyzed using Microsoft Excel 2021 for descriptive statistics. The results were presented as frequencies, percentages, and DDD values to describe patterns of antibiotic use and DRP distribution. This study obtained ethical approval from the Research Ethics Committee of Airlangga University Hospital (No. 115/KEP/2023).

## 3 Results

**Fig. 1:**
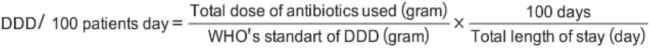
DDD formula.^3^

Table 4 shows patients characteristics, including gender, age, length of hospital stay, type of disease, and culture examination results. Among 125 patients, there were 65 male patients (52 %), which was higher than the number of female at 60 (48 %). The majority of patients were adults aged 18–59 years, accounting for 63 patients (50 %). The most common length of hospital stay was 6–10 days (30 %), with an average duration of 16 days. Furthermore, the most prevalent disease classification was pneumonia (40 %), followed by sepsis and urinary tract infection. (See Fig. 1.) (See Table 1, Table 2, Table 3.)

**Table 1:** Classification of DRPs according to Cipolle.

Primary Domain	Classification Cipolle
Indication	1. Unnecessary drug therapy 2. Needs additional drug therapy
Effectiveness	1. Dosage too low 2. Ineffective drug
Drug Safety	1. Dosage too high 2. Adverse drug reaction
Noncompliance	Noncompliance

**Table 2:** PCNE classification of DRPs - Problem (P).

Primary Domain	PCNE Problem (P)
1. Treatment effectiveness There is a (potential) problem with the (lack of) effect of the pharmacotherapy.	P1.1 – No effect of drug treatment despite correct use P1.2 – Effect of drug treatment not optimal P1.3 – Untreated symptoms or indication
2. Treatment safety Patient suffers, or could suffer, from an adverse drug event.	P2.1 – Adverse drug event (possibly) occurring
3. Other	P3.1 – Unnecessary drug-treatment P3.2 – Unclear problem

**Table 3:** PCNE classification of DRPs - Cause (C).

Primary Domain	PCNE Cause (C)
1. Drug selection The cause of the (potential) DRP is related to the selection of the drug (by patient or health professional)	C1.1 – Inappropriate drug according to guidelines/formulary C1.2 – No Indication for drug C1.3 – Inappropriate combination of drugs, or drugs and herbal medications, or drugs and dietary supplements C1.4 – Inappropriate duplication of therapeutic group or active ingredient C1.5 – No or incomplete drug treatment in spite of existing indication C1.6 – Too many different drugs/active ingredients prescribed for indication
2. Drug form The cause of teh DRP is related to the selection of the drug form	C2.1 – Inappropriate drug form/formulation (for this patient)
3. Dose selection The cause of the DRP is related to the selection of the dose or dosage	C3.1 – Drug dose too low C3.2 – Drug dose of a single active ingredient too high C3.3 – Dosage regimen not frequent enough C3.4 – Dosage regimen too frequent C3.5 – Dose timing instructions wrong, unclear or missing
4. Treatment duration The cause of the DRP is related to the duration of treatment	C4.1 – Duration of treatment too short C4.2 – Duration of treatment too long
5. Dispensing The cause of the DRP is related to the logistics of the prescribing and dispensing process	C5.1 – Prescribed drug not available C5.2 – Necessary information not provided or incorrect advice provided C5.3 – Wrong drug, strength or dosage advised (OTC) C5.4 – Wrong drug or strength dispensed
6. Drug use process The cause of the DRP is related to the way the patient gets the drug administered *by a health professional or other carer*, despite proper dosage instructions (on label/list)	C6.1 – Inappropriate timing of administration or dosing intervals by a health professional C6.2 – Drug under-administered by a health professional C6.3 – Drug over-administered by a health professional C6.4 – Drug not administered at all by a health professional C6.5 – Wrong drug administered by a health professional C6.6 – Drug administered via wrong route by a health professional
7. Patient related The cause of the DRP is related to the patient and his behaviour (intentional or non- intentional)	C7.1 – Patient intentionally uses/takes less drug than prescribed or does not take the drug at all for whatever reason C7.2 – Patient uses/takes more drug than prescribed C7.3 – Patient abuses drug (unregulated overuse) C7.4 – Patient decides to use unnecessary drug C7.5 – Patient takes food that interacts C7.6 – Patient stores drug inappropriately C7.7 – Inappropriate timing or dosing intervals C7.8 – Patient unintentionally administers/uses the drug in a wrong way C7.9 – Patient physically unable to use drug/form as directed C7.10 – Patient unable to understand instructions properly
8. Patient transfer related The cause of the DRP can be related to the transfer of patients between primary, secondary and tertiary care, or transfer within one care institution.	C8.1 – Medication reconciliation problem
9. Other	C9.1 – No or inappropriate outcome monitoring (incl. TDM) C9.2 – Other cause; specify C9.3 – No obvious cause

The administration of antibiotic in ICU of Universitas Airlangga Hospital was classified into single and combination, as shown in Table 5. The most frequently used single antibiotic was ceftriaxone, administered to 51 patients (47 %), for upper respiratory tract infections, followed by moxifloxacin (22 %) and levofloxacin (20 %). The most common antibiotic combination was ceftriaxone with metronidazole, accounting for 5 % of cases for infections including both aerobic and anaerobic bacteria.

During the period of July–December 2022, the antibiotic therapy data showed that the highest DDD per 100 patient-days was for ceftriaxone, with a value of 31.57 DDD/patient-days. This showed that approximately 32 patients received ceftriaxone at the WHO-recommended dose of 2 g.

Table 6 showed the classification of DRPs in antibiotic use based on the Cipolle method. Based on the results, the most frequently identified problems were unnecessary drug therapy, which occurred in 20 % of cases. This showed that antibiotic was administered without a clear clinical indication. The second most common issue was ineffective drug therapy (16 %), suggesting that antibiotic used was not the most appropriate option for the pathogens, or alternative agents could have provided better therapeutic outcomes. Additionally, dose too low was observed in 5 % of cases, while too high occurred in 3 %, reflecting inaccuracies. Adverse drug reactions were reported in 1 % of patients, showing a relatively low but significant incidence of antibiotic-related side effects. These results showed the need for improved antibiotic stewardship, particularly in ensuring appropriate indication, selection, and dosing to minimize unnecessary or ineffective antibiotic use.

Table 7 showed the classification of the causes of antibiotic use problems according to PCNE in the Cause (C) section. The most dominant cause was found to be the absence of a medical indication for antibiotic administration (C1.2 – No indication for drug) at 20 %, followed by the selection of drugs that did not comply with guidelines or formulary (C1.1 – Inappropriate drug according to guidelines/formulary) at 15 %. Other causes identified were the duration of therapy being too short (C4.1 – Duration of treatment too short) at 5 %, the dose being too low (C3.1 – Drug dose too low) at 2 %, the administration interval being inappropriate (C3.3 – Dosage regimen not frequent enough) at 2 %, and the duration of therapy too long (C4.2 – Duration of treatment too long) at 1 %. These results showed that most issues originated from inappropriate indications and antibiotic selection, requiring interventions to improve the rationality of prescriptions.

Table 8 showed the classification of the causes of antibiotic use problems according to PCNE in the Cause (C) section. Based on the results, the most dominant cause was the absence of a medical indication for antibiotic administration (C1.2 – No indication for drug) at 20 %, followed by the selection of drugs that did not comply with guidelines or formulary (C1.1 – Inappropriate drug according to guidelines/formulary) at 15 %. Other causes identified were too short duration of therapy (C4.1 – Duration of therapy too short) at 5 %, the dose being too low (C3.1 – Drug dose too low) at 2 %, the administration interval being inappropriate (C3.3 – Dosage regimen not frequent enough) at 2 %, and the duration of therapy being too long (C4.2 – Duration of treatment too long) at 1 %. These results showed that most issues occurred from inappropriate indications and antibiotic selection, requiring interventions to improve the rationality of prescriptions.

## 4 Discussion

The rational use of antibiotic can be carried out as a prevention of resistance, which has become a global issue in increasing morbidity and mortality rates. Therefore, this study examined the quantitative and qualitative use of antibiotic through DDD and Gyssens methods, respectively. The samples used were hospitalized patients in ICU to assist the KPRA program in optimizing the wise use of antibiotic for preventing resistance. The results of DDD value showed the amount of antibiotic use in accordance with WHO regulations, measured in DDD/100 patient-days. Meanwhile, the quality analysis used the Gyssens method to determine and evaluate the rationality of antibiotic use in hospital. This descriptive study used retrospective data obtained from the medical records room of RS Universitas Airlangga, Surabaya. Data were obtained through total sampling of ICU patients who met the inclusion criteria, covering the period from July 1 to December 31, 2022. A total of 125 adult patients over 18 years met the inclusion criteria, comprising 52 % male and 48 % female (Table 4).

**Table 4:** Patients characteristics.

Patients Characteristics	Number	Percentage (%)
Gender		
Male	65	52
Female	60	48

Age		
18–59 Years	62	50
>60 Years	63	50

Length of Hospital Stay		
1–5 days	16	13
6–10 days	35	30
11–15 days	32	27
16–20 days	10	8
21–25 days	15	13
26–30 days	5	4
>30 days	2	1
Average Length of Hospital Stay	16	–

Type of Disease		
Pneumonia	64	40
Sepsis	41	25
Urinary Tract Infection	18	11
Tuberkulosis	10	6
Diabetic Foot Infections	5	3
COPD	5	3
Pressure Ulcer	5	3
Gastroenteritis	3	2
Aspiration Pneumonia	3	2
HIV	2	1
ISPA	2	1
Meningoensefalitis	2	1
LRTI	1	1

Culture Examination		
Examined	53	42
Not Examined	72	58

The distribution of data based on gender was consistent with the study by Lat, T.I,^15^ which reported similar results regarding the role of reproductive hormones in the immune response in critical disease. The reproductive hormone estrogen in women is suspected to be immunoprotective after severe trauma and blood loss, as well as in sepsis conditions. Then, androgen hormones have been proven to suppress the immune system.^15^ Another study stated that the number of male patients in ICU was higher, with greater mortality rate due to infectious diseases of the respiratory system, such as tuberculosis, COPD, and CAP.^16^

Based on age, the highest prevalence of ICU patients was found in the elderly group over 60 years old, accounting for 50 % (Table 4). This result was consistent with another study, where the average age of ICU patients was 66 years.^17^ Meanwhile, patients aged 65–84 years and over 85 years have been found to experience higher hospitalization rates and mortality.^18^ There is a similar statement reporting that patients over 65 years old are more vulnerable to death because of the decrease in body system functions. According to the 2018 Riskesdas Indonesia data, the incidence of pneumonia increases with age, reaching 2.5 %, 3.0 %, and 2.9 % in 55–64, 65–74, and 75 years, respectively.^19^

Advancing age can lead to a decrease in physiological reserves that play a role in maintaining body balance during disease.^16^ Physiological changes that show age-related immune dysregulation and changes in respiratory capacity include loss of lung elasticity, decreased muscle strength, and altered sensitivity of the respiratory center to hypoxia and/or hypercapnia. This contributes to a high risk of vulnerability to pathogens, viral reactivation, respiratory failure, and death.^20^ A previous study stated that increasing age was often associated with changes in the body's immune function, such as the decreased ability of leukocytes to eliminate antigens and changes in the expression of pro-inflammatory cytokines.^21^

The prevalence of Length Of Stay (LOS) for ICU patients is highest in the 6 to 10-day classification, with 35 patients (30 %), as shown in Table 4. In this study, the total LOS is 1411 days, calculated from the total days of all patients. The LOS for patients with a prevalence of 6 to 10 days is based on condition stability and related to the duration of antibiotic administration alongside monitoring during hospital care. The shortest LOS is found in the 1 to 5-day category, with 16 patients (13 %). Relatively short LOS is observed in patients who died when receiving therapy in ICU, as well as those with good clinical condition improvement. Moreover, previous studies had reported a positive relationship between infection rates and LOS because high-risk patients could contract nosocomial infections and often harbor antibiotic-resistant microorganisms.^22^, ^23^, ^24^, ^25^, ^26^ Several factors that influence LOS include patients characteristics, clinical conditions, medical procedures received, management, and hospital administration.^27^

The profile of infectious diseases in patients receiving antibiotic in ICU of RS Universitas Airlangga is presented in Table 4, with the most prevalent being pneumonia at 40 %, sepsis 27 %, and urinary tract infections 11 %. Pneumonia includes infections such as Community-acquired pneumonia (CAP), Hospital-Acquired Pneumonia (HAP), Ventilator-Associated Pneumonia (VAP), and bacteria pneumonia caused by COVID-19.

One of the core strategies in the management of antimicrobial in hospital is to implement prescription regulations. This process considers that antimicrobial is grouped into the AWaRe categories, namely access, watch, and reserve, to control use based on the authority established by the hospital leadership.^28^ AWaRe classification is expected to reduce the rate of AMR by creating policies for monitoring antibiotic consumption. Additionally, it helps policymakers relate each country's essential drug list with the WHO Model to curb the consumption of watch and reserve antibiotic.^29^

The administration of antibiotic to patients with bacteria infections can be given empirically and definitively based on culture test results. In this study, the profile of antibiotic use in patients showed the highest results in ceftriaxone at 47 % and the combination of ceftriaxone and metronidazole at 5 %. These results were consistent with the report by Putri et al. in ICU of Soedarso Hospital, Pontianak, from January to June 2019. According to the report, the most commonly used single antibiotic was ceftriaxone, and the combination antibiotic were ampicillin sulbactam with metronidazole, cefadroxil with cefotaxime, and ceftriaxone with metronidazole.^30^

Watch group is used as therapy under special conditions or when the administration of the access group is no longer effective. There is a meta-analysis regarding the risk of double colonization by antibiotic-resistant pathogens found in watch classification compared to unexposed pathogens.^31^ Based on these results, stricter monitoring of watch group is essential to secure the efficacy of antibiotic administration.^29^ Another reason for tightening the use of watch group is a higher potential to become resistant, making it a primary target in surveillance and monitoring programs.^32^

The evaluation of antibiotic use quantity through DDD method is one of the core strategies in the PGA at hospital. The quantity of antibiotic use at RS Universitas Airlangga in Table 5 showed a total of 91.54 DDD/patient-days. This showed that in 100 patients days, there were 92 patients receiving antibiotic at dose in accordance with WHO standards. Ceftriaxone (IV) was the antibiotic with the highest DDD value at 31.57 DDD/patient-days. The data showed that approximately 32 patients received ceftriaxone at the dose recommended of 2 g by WHO.

**Table 5:** Results of quantitative analysis of the DDD method in ICU of Airlangga University Hospital.

Antibiotic Class	Antibiotic Name	ATC Code	Number of Uses (n patients)	DDD WHO (gram)	Total Antibiotic Use (gram)	Total DDD	LOS	Total DDD/100 days	Category aware
Cephalosporins	Ceftriaxone	J01DD04	71	2	891	445.5	1411	31.57	Watch
Fluoroquinolones	Levofloxacin	J01MA12	30	0.5	124.5	249	1411	17.65	Watch
Floroquinolon	Moxifloxacin	J01MA14	29	0.4	70.4	176	1411	12.47	Watch
Nitroimidazoles	Metronidazole	J01XD01	10	1.5	111	74	1411	5.24	Access
Cephalosporins	Ceftazidime	J01DD02	13	4	258	64.5	1411	4.57	Watch
Penicillins	Ampicillin–Sulbactam	J01CA01	10	6	381	63.5	1411	4.50	Access
Floroquinolon	Ciprofloxacin (IV)	J01MA02	4	0.5	25.6	51.2	1411	3.63	Watch
Sulfonamides-Trimethoprim	Sulfamethoxazole–Trimethoprim	J01EC01	3	2	89.28	44.64	1411	3.16	Access
Aminoglycosides	Amikacin	J01GB06	6	1	36	36	1411	2.55	Watch
Carbapenems	Meropenem	J01DH02	6	3	101	33.67	1411	2.39	Reserve
Fluoroquinolones	Ciprofloxacin (PO)	J01MA02	3	1	17	17	1411	1.20	Access
Cephalosporins	Cefprozil	J01DC10	1	1	9	9	1411	0.64	Watch
Cephalosporins	Cefoperazone–Sulbactam	J01DD12	3	4	33	8.25	1411	0.58	Watch
Cephalosporins	Cefixime	J01DD08	2	0.4	2.4	6	1411	0.43	Watch
Cephalosporins	Cefotaxime	J01DD01	1	4	24	6	1411	0.43	Watch
Aminoglycosides	Streptomycin	J01GA01	1	1	3	3	1411	0.21	Access
Macrolides	Azithromycin	J01FA10	1	0.3	1.5	3	1411	0.21	Watch
Glycopeptides	Vancomycin	J01XA01	1	2	4	1.33	1411	0.09	Reserve
**Total**								**91.54**	

Antibiotic with the highest quantity in this study is identical to other reports, showing DDD of ceftriaxone at 35.79 DDD/100 patient-days in ICU patients at RS Muhammadiyah Malang in 2022.^33^ Another study at RS Islam Jakarta Pondok Kopi in 2020 found that antibiotic with the highest DDD value was ceftriaxone, with a value of 39.80 DDD/patient-days.^34^ In a study at RS Dr. Soedarso Pontianak, Indonesia in 2019, the most commonly used antibiotic in ICU patients was ceftriaxone, with DDD value of 76.15 DDD/100 patient-days.^35^ A study conducted at RSUD Provinsi NTB in 2018 found that ceftriaxone had the highest value of 60.71 DDD/patient-days, followed by levofloxacin with 29.15 DDD/patient-days, and meropenem at 16.10 DDD/patient-days.^36^

According to WHO dose recommendations, a previous study stated that the administration of ceftriaxone at a dose of 2 g was more recommended compared to 1 g for patients in ICU with a lower risk of death.^37^ Ceftriaxone is an empirical antibiotic widely known for many ICU patients, particularly those with pneumonia and/or urinary tract infections. Third-generation cephalosporins have lower sensitivity to most gram-positive organisms but broader coverage against Enterobacteriaceae, Neisseria spp., and *H. influenzae.*^38^ In this study, the use of ceftriaxone was frequently prescribed for indications of pneumonia and urinary tract infections. Ceftriaxone is widely prescribed to patients in ICU because of high pharmacokinetic profile with renal and biliary excretion. There is also a relatively long half-life of 6 to 8 h, thereby not requiring dose adjustments in patients with renal failure. Fluoroquinolones in group III (levofloxacin) and group IV (moxifloxacin) have good activity against Gram-positive and Gram-negative bacteria.^39^ Based on the PPAB RSUA in 2022, moxifloxacin was administered to patients with renal impairment or those previously given levofloxacin without improvement. Moxifloxacin is often given to patients with kidney disease because only 20 % of drug is excreted unchanged by kidneys, without requiring dose adjustment.^39^

The classification of DRPs related to antibiotic use according to the Cipolle framework is presented in Table 6. Based on the results, the most commonly identified problems included **“**Unnecessary drug therapy**”** (20 %), showing that antibiotic was prescribed without a valid clinical indication. This shows a pattern of overuse that can contribute significantly to the development of antimicrobial resistance, particularly in intensive care settings where empirical therapy is frequently used.40., 41

**Table 6:** Classification of DRPs antibiotic use according to Cipolle.

Problems Description	Cipolle Classification	Percentage
No indication for antibiotic	Unnecessary drug therapy	20 %
Antibiotic use too short	Dosage too low	5 %
Antibiotic use not at the correct dosage	Ineffective drug	16 %
There are other antibiotic that are more effective	Ineffective drug	
There are other antibiotic with a narrower spectrum	Ineffective drug	
There are other antibiotic that are safer	Adverse drug reaction	1 %
Antibiotic administration interval not correct	Dosage too high	3 %
Antibiotic use too long	Dosage too high	

Another dominant issue was the classification of **“**Ineffective drug**”**, contributing to 16 % of DRPs. This category included cases where a more effective antibiotic was available, use of broader or narrower spectra, or selection of an inappropriate antibiotic for the type of infection. The results showed a gap in individualized antibiotic selection, which could lead to treatment failure or delayed recovery.^42^

Dosing-related problems were prevalent, with “Dosage too low” reported in **5 %** and “Dosage too high” in 3 % of cases. Generally, incorrect dosing can lead to subtherapeutic concentrations, promoting bacteria resistance or toxicity that increases the risk of adverse outcomes.^43^ Inappropriate administration intervals (3 %) further suggest a need for closer adherence to pharmacokinetic and pharmacodynamic principles, particularly among patients with chronic disease.^44^

Adverse drug reactions were reported in 1 % of cases, showing the importance of routine monitoring and careful antibiotic selection, specifically in high-risk populations such as ICU patients. These results show the need for strengthened AMS interventions in ICU settings. To better contextualize local data, future discussions should incorporate international AMS benchmarks, such as AWaRe classification and the Global Antimicrobial Resistance and Use Surveillance System (GLASS). The data would be applied to compare antibiotic use rates and resistance patterns across different healthcare systems.^3^,^11^

The discussion can be enhanced by including visual or statistical comparisons such as graphical shows of DDD values across antibiotic classes. This will facilitate interpretation and engagement for clinicians and policymakers.^45^

Since methodological transparency is essential, this study provides some important recommendations. These include an in-depth description of how DRPs are identified, validated, and reviewed, alongside the use of Cipolle and PCNE v9.1 criteria to ensure consistency and reproducibility.^40^ Strengthening interdisciplinary collaboration, particularly between clinical pharmacists, microbiologists, and infectious disease specialists, can further reduce inappropriate antibiotic use and improve patients outcomes.^3^,^12^

The classification of antibiotic-related DRPs based on the PCNE system in terms of the problems identified (Problem/P) is shown in Table 7. Based on the results, the most frequently identified issue was P1.2 – Effect of drug treatment not optimal (23 %), showing that the therapeutic effect of the administered antibiotic was suboptimal. The phenomenon can be due to incorrect dosing, inappropriate administration intervals, or the selection of less suitable antibiotic agents. P3.1 – Unnecessary drug-treatment (21 %) was also identifed, showing the use of antibiotic without a clear medical indication, which increased the risk of antibiotic resistance.^46^

**Table 7:** Classification of DRP antibiotic use according to PCNE (Problem/P).

Problems Description	PCNE Problems (P)	Percentage
No indication for antibiotic	P3.1 – Unnecessary drug therapy	21 %
Prolonged use of antibiotic	P3.1 – Unnecessary drug therapy	
Shortened use of antibiotic	P1.2 – Effect of drug therapy not optimal	23 %
Incorrect dosage of antibiotic	P1.2 – Effect of drug therapy not optimal	
Incorrect interval of antibiotic administration	P1.2 – Effect of drug therapy not optimal	
There are other antibiotic that are more effective	P1.2 – Effect of drug therapy not optimal	
There are other antibiotic that are safer	P2.1 – Adverse drug event (possibly) occurring	1 %
There are other antibiotic with a narrower spectrum	P2.1 – Adverse drug event (possibly) occurring	

Other issues were observed in smaller proportions, such as adverse drug events (P2.1) occurring in 1 % of cases and the use of antibiotic when more effective or safer alternatives were available. The results show the need for regular evaluation of antibiotic regimens to ensure necessity and efficacy in treatment.

Table 8 provides an analysis of the causes (Cause/C) of antibiotic-related DRPs. The most frequently reported cause was C1.1 –Inappropriate drug according to guidelines/formulary (15 %), suggesting that the selected antibiotic did not comply with established treatment guidelines or the hospital formulary. This underlines the importance of up-to-date clinical protocols and the consistent implementation. Additionally**,** C1.2 – No indication for drug (20 %) showed that antibiotic was prescribed without a valid clinical indication, supporting the results from Table 8 (P3.1).

**Table 8:** Classification of DRP antibiotic use according to PCNE (Cause/C).

Problems Description	PCNE Cause (C)	Percentage
No indication for antibiotic	C1.2 – No Indication for drug	20 %
Prolonged use of antibiotic	C4.2 – Duration of treatment too long	1 %
Shortened use of antibiotic	C4.1 – Duration of treatment too short	5 %
Incorrect dosage of antibiotic	C3.1 – Drug dose too low	2 %
Incorrect interval of antibiotic administration	C3.3 – Dosage regimen not frequent enough	2 %
There is another antibiotic that is more effective	C1.1 – Inappropriate drug according to guidelines/formulary	15 %
There is another antibiotic that is safer	C1.1 – Inappropriate drug according to guidelines/formulary	
There is another antibiotic with a narrower spectrum	C1.1 – Inappropriate drug according to guidelines/formulary	

Other significant causes included overly long (C4.2, 1 %) or too short (C4.1**,** 5 %) duration of therapy, incorrect dosage (C3.1, 2 %), and inappropriate intervals of administration (C3.3, 2 %). These results showed gaps in clinical judgment and antibiotic selection, which could be mitigated through clinical pharmacist intervention and robust AMS programs (Pulcini et al., 2019).

Compared to previous studies, the predominance of ceftriaxone use in this ICU correlated with reports from other Indonesian hospitals and regional data in Asia.^35^,^36^ However, the DDD values (31.57 DDD/100 patient-days) were slightly lower than some tertiary hospitals, suggesting potential differences in antibiotic stewardship implementation or case severity.^33^

The relatively high rate of DRPs, particularly unnecessary antibiotic therapy and suboptimal dosing, shows continuous challenges in rational prescribing. Similar issues have been documented globally, emphasizing the importance of integrating pharmacy-led interventions and continuous audit-feedback mechanisms within ICU AMS programs.^11^,^45^ Clinically, these results show that high guideline adherence, optimized dosing strategies, and timely culture-based therapy can substantially reduce DRP rates and improve patients outcomes. Regular staff education and collaboration between physicians and pharmacists are also critical for sustaining stewardship effectiveness.^3^

This study has several limitations that should be acknowledged. Due to its retrospective design relies on the completeness and accuracy of medical records, which may introduce information bias.

Despite these limitations, the results provide practical insights for optimizing antibiotic stewardship in ICU settings. The observed trends in DDD and DRPs can guide hospital antimicrobial committees in developing targeted interventions, such as empirical therapy guidelines, antibiotic restriction policies, and pharmacist-driven reviews, to promote rational antibiotic use and minimize resistance development. Moreover, future multicenter studies incorporating clinical outcomes and microbiological data are recommended to strengthen the results and inform evidence-based stewardship strategies.^3^,^12^

## 5 Conclusion

In conclusion, this study shows that antibiotic use in ICU of Airlangga University Hospital is dominated by ceftriaxone, with the highest consumption rate of 31.57 DDD per 100 patient-days. Analysis of DRPs using the Cipolle and PCNE v9.1 classifications shows that the most prevalent issues are unnecessary antibiotic therapy and suboptimal therapeutic outcomes, primarily caused by inappropriate indications and deviations from established treatment guidelines.

The results show that despite existing AMS policies, the rational use of antibiotic in critical care remains suboptimal. The high incidence of DRPs shows the urgent need for strengthened stewardship interventions focusing on evidence-based prescribing, adherence to local and international guidelines, alongside consistent monitoring of antibiotic consumption through DDD metrics.

To enhance therapeutic effectiveness and reduce antimicrobial resistance, interdisciplinary collaboration among clinicians, pharmacists, and microbiologists should be prioritized. Routine audits, feedback mechanisms, and integration of culture-based therapy are essential to ensure the appropriateness of antibiotic selection and dosing. Moreover, future prospective studies incorporating microbiological and clinical outcome data are recommended to validate these results and support the development of sustainable, data-driven AMS programs in ICU settings.

## CRediT authorship contribution statement

**Yulistiani Yulistiani:** Supervision, Project administration, Methodology, Data curation, Conceptualization. **Hargus Haraudi Barkah:** Writing – review & editing, Supervision, Data curation. **Febriansyah Nur Utomo:** Writing – review & editing, Supervision, Methodology. **Lucky Andrianto:** Validation, Supervision, Formal analysis, Data curation, Conceptualization. **Alena Putri Jathi:** Writing – review & editing, Writing – original draft, Investigation, Formal analysis, Data curation. **Nurrizky Grahitaning Putra Rohmaana Yudistira:** Writing – original draft, Data curation.

## Funding

There are no fundings for this research.

## Declaration of competing interest

The authors declare the following financial interests/personal relationships which may be considered as potential competing interests:

Yulistiani reports administrative support was provided by Airlangga University Faculty of Pharmacy. Yulistiani reports a relationship with Airlangga University Faculty of Pharmacy that includes: employment. Yulistiani has patent pending to -. Corresponding Author, Yulistiani had no involvement in the peer-review of this article and has no access to information regarding its peer-review. Full responsibility for the editorial process for this article was delegated to another journal editor. If there are other authors, they declare that they have no known competing financial interests or personal relationships that could have appeared to influence the work reported in this paper.
